# Upper Limb Portable Motion Analysis System Based on Inertial Technology for Neurorehabilitation Purposes

**DOI:** 10.3390/s101210733

**Published:** 2010-12-02

**Authors:** Rodrigo Pérez, Úrsula Costa, Marc Torrent, Javier Solana, Eloy Opisso, César Cáceres, Josep M. Tormos, Josep Medina, Enrique J. Gómez

**Affiliations:** 1 Bioengineering and Telemedicine Centre, ETSI Telecomunicación, Universidad Politécnica de Madrid, 28040, Madrid, Spain; E-Mails: jsolana@gbt.tfo.upm.es (J.S.); ccaceres@gbt.tfo.upm.es (C.C.); egomez@gbt.tfo.upm.es (E.J.G.); 2 Institut Guttmann Neurorehabilitation Hospital, 08916, Barcelona, Spain; E-Mails: ucosta@guttmann.com (U.C.); eopisso@guttmann.com (E.O.); jmtormos@guttmann.com (J.M.T.); jmedina@guttmann.com (J.M.); 3 CETEMMSA Research Centre, 08301, Mataró, Spain; E-Mail: mtorrent@cetemmsa.com (M.T.)

**Keywords:** motion tracking, inertial sensors, neurorehabilitation, upper limb, biomechanical model

## Abstract

Here an inertial sensor-based monitoring system for measuring and analyzing upper limb movements is presented. The final goal is the integration of this motion-tracking device within a portable rehabilitation system for brain injury patients. A set of four inertial sensors mounted on a special garment worn by the patient provides the quaternions representing the patient upper limb’s orientation in space. A kinematic model is built to estimate 3D upper limb motion for accurate therapeutic evaluation. The human upper limb is represented as a kinematic chain of rigid bodies with three joints and six degrees of freedom. Validation of the system has been performed by co-registration of movements with a commercial optoelectronic tracking system. Successful results are shown that exhibit a high correlation among signals provided by both devices and obtained at the Institut Guttmann Neurorehabilitation Hospital.

## 1. Introduction

The World Health Organization (WHO) predicts that by the year 2020, acquired brain injury (ABI) will be among the ten most common ailments. These injuries dramatically change the life of the patients and their families due to their physical, sensory, cognitive, emotional and socio-economic consequences. The cause of ABI can be either traumatic (car accidents, falls, *etc*.) or non-traumatic (strokes, brain tumors, infections, *etc*.). The most common ABIs are stroke and traumatic brain injury (TBI) [1].

Every year, nine million people around the world suffer from stroke [2]. Globally, cerebrovascular disease (stroke) is the second leading cause of death and the eighth cause of severe disability in the elderly. The WHO estimated that in 2005, stroke accounted for 5.7 million deaths worldwide, equivalent to 9.9% of all deaths, and was the predominant cause of disability, afflicting 30.7 million people [2]. Statistical data show that after a stroke, one third of patients die during the first month, and 40% of people who recover from the acute phase exhibit a high degree of impairment that decreases their independence. Only one third of patients recover their basic functions and can resume a normal life [3]. There are not accurate data on the prevalence of TBI in Europe, meanwhile data from the United States show a high prevalence of this pathology, with 5.3 million people living with a disability from TBI [4].

New techniques of early intervention and the development of intensive ABI care have noticeably improved the survival rate. However, in spite of these advances, brain injuries still have no surgical or pharmacological treatment to re-establish lost function. Neurorehabilitation therapies address this problem by restoring, minimizing or compensating the functional alterations in a person disabled because of a nervous system injury. Medical evidence in neurorehabilitation is scarce and the assessment methods, especially those dealing with upper limb function, depend on clinician experience and subjectivity. Moreover, motion analysis assessments, which are more sensitive and provide objective data, are mainly centered on gait analysis, whereas upper limb tests are still not widely performed. Current upper limb motion assessments in neurologic population are focused on single-joint kinematics. Moreover, clinical tests are highly dependent on the examiner criteria. Further development of reliable and valid multi-joint biomechanical evaluations is required, particularly for goal oriented reaching movements [5]. The lack of standardized protocols due to the large variety of movements, complexity of the upper extremity and lack of international consensus to validate the protocols hampered the advance on this area [6].

Many attempts have been done to evaluate upper limb kinematics in neurologic population. Typically, these motion analyses are focused on the study of analytical tasks [7]. Moreover, current 3D kinematic models include sacrum or pelvic markers [8]. This might jeopardize the application of these models in neurologic population due to pelvic instability and lack of trunk control.

Some advances occurred in the last five years with the publication of normal values during functional tasks in adults [6,9,10]. Nevertheless, protocols used in these studies include pelvic markers, hampering the application in neurologic population.

One of the major objectives of neurorehabilitation is to provide patients with the capacity to perform specific activities of the daily life (ADL) required for an independent life. Recently, research has commonly addressed measurements of upper limb movements because these limbs are frequently used to contact and manipulate objects [11]. Functional assessments based on motion tracking of ADL are needed to create new knowledge and increase the efficiency of ABI neurorehabilitation.

Devices that accurately track human motion are a key component of current physical rehabilitative systems. These devices allow therapists to record movements performed by their patients so that they can remotely study the results. There are many commercial systems that make human motion tracking feasible. These systems use different sensor technologies, including electromagnetic, visual, mechanical and inertial sensors. It is important to note that currently, these systems are almost exclusively used for gait analysis. Upper limb motion analysis is still being developed.

BTS SMART-D [12] and Vicon MX [13] are the most popular general-purpose high-precision optoelectronic digital systems for gait analysis. These systems basically consist of a set of infrared cameras directly connected to an integration box that contains appropriate software for data capture, 3D reconstruction, motion analysis and visualization of human movements. Although these two systems are well suited to the task of motion analysis, their complexity and characteristics make them infeasible for integration into a portable real-time rehabilitative system. Photogrammetry-based systems [14] use video cameras as acquisition devices to monitor how different body parts move. A good example of this type of system is the Kinescan [15].

Electromagnetic motion capture systems [16] are an alternative to those previously described. These systems have been widely used for tracking human movements in virtual reality due to their small size, high sampling rate and precision. The main advantage of these systems is the absence of marker occlusions because the electromagnetic signal is always “seen” by the receiver within a maximum distance. Examples of magnetic motion capture systems are MotionStar [17] and LIBERTY [18]. On the other hand, one of the main weaknesses of these systems is the latency and jitter that arise due to the nature by which sensor measurements are conducted [19]. These disadvantages, along with possible interferences that can take place in uncontrolled environments, make electromagnetic systems not suitable for portable rehabilitative systems.

Inertial measurements [20] are a technique designed to measure and report the orientation and velocity of an object without the need of an external reference. Inertial Measurement Units (IMUs) are based on the use and combination of different inertial sensor technologies, including accelerometers, gyroscopes and magnetometers, to provide an accurate estimation of orientation referenced to a fixed frame. Gyroscopes provide a measurement of the angular velocity applied to the object and thus an estimation of the rotated angle and actual orientation if an initial reference is provided. Because gyroscopes have different sources of dynamic drift, the estimation of orientation deteriorates with time. To correct these effects, accelerometers and magnetometers are added to the system through data fusion algorithms so that external references are provided for drift correction. Accelerometers give a measure of the direction of the gravity vector, and magnetometers provide measurements of the direction of the Earth’s magnetic field. With this technology, IMUs are able to accurately estimate their own orientation with respect to a fixed reference frame formed by gravity and the Earth’s magnetic North vectors.

In the field of motion capture, IMUs can be used to provide continuous orientation data from unrestricted movements when the devices are correctly mounted on a subject. Because these sensors are sourceless, compact and light, they have become an interesting choice for portable motion tracking applications [21–24]. The FAB system [25] is a commercial system based on the application of inertial technology to motion acquisition. This general-purpose system is able to monitor in real time different human movements regardless of the limb used. A full review of human motion tracking systems for rehabilitation can be found in [26].

The main goal of the present research was to develop a real-time portable upper limb motion acquisition system for upper limb DLAs and to perform a preliminary validation of the applied methodology so as to provide therapists with measurements of the motions performed by their patients so that an accurate and personalized therapeutic evaluation can be carried out. For this purpose, a kinematic model and a degrees of freedom (DoF) calculation methodology was created. The monitoring device proposed in this paper is also intended to work within a virtual reality (VR)-based rehabilitation system to provide the user with visual feedback about the upper limb position and performance relative to a certain established ADL pattern.

This paper is organized as follows. Section 2 describes the whole inertial technology-based motion tracking system, from the defined biomechanical model to the methodology applied to calculate the different upper limb degrees of freedom, and the experimental work carried out for the system validation. Section 3 shows the obtained results, and finally, Section 4 states the conclusions extracted from this work.

## 2. Methods

### 2.1. System Description

In this research, a portable motion capture system based on inertial measurement is proposed. For this purpose, commercially available MTi Xsens inertial sensing units have been used [27]. These miniaturized sensors include a combination of inertial sensors and an embedded processor to calculate an absolute orientation estimation (roll, pitch and yaw), acceleration, angular velocity and magnetic North in real time.

**Figure 1.**
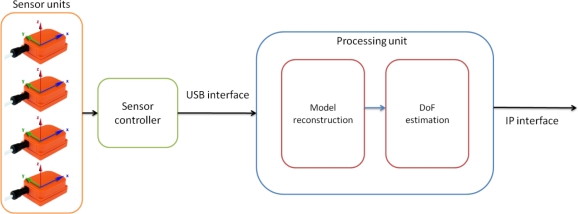
Schematic view of the motion capture system.

**Figure 2.**
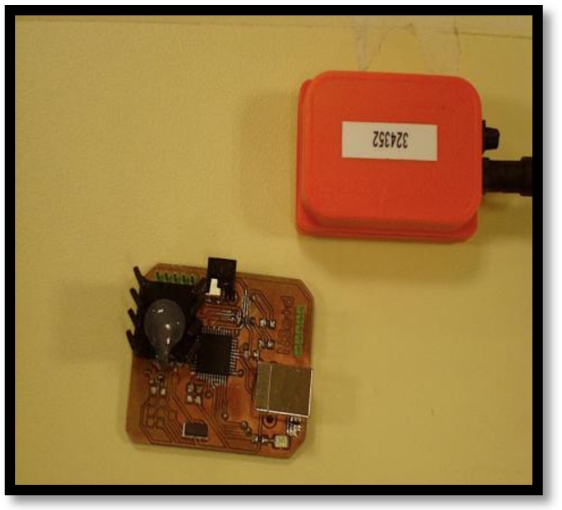
System integration box.

The motion capture system presented in this research work (Figure 1) is composed of four MTi sensing units strategically placed on the upper limb. All of the units are controlled by a sensor controller device designed specifically for this application. The units and the controller (Figure 2) communicate through a 4-wire data and power bus in such a way that the data from the units are sent to a PC via the controller using a standard USB connection. This sensor controller has been specifically designed and built to control the inertial sensing units; its main functionalities are the configuration of the units, their calibration, the synchronization of the data channel between the sensors and the computer, and the error management. An automatic configuration sequence is launched by the controller each time the system is powered up. This operation is applied to each sensor unit to configure the sensors for the application. The controller applies a sequence for the calibration of the units when needed, manages the communication among the sensing units and maintains a perfect synchronization of all sensor units to ensure minimal data loss and a good performance. The third functionality of the controller is to ensure a proper recovery of the system, if possible, when a failure takes place.

The present work mainly uses rotation matrices [28] to perform different calculations. These matrices, that represent the sensors 3D orientation, are provided by Xsens MTi inertial sensors following (1), where *ψ* is the rotation about the z-axis (yaw), *θ* is the rotation about the y-axis and *φ* is the rotation about the z-axis (roll):

(1) $$R=R_{\psi }^{Z}\cdot R_{\theta }^{Y}\cdot R_{\phi }^{X}=\left[\begin{array}{ccc} \text{cos}\psi & -\text{sin}\psi & 0\\ \text{sin}\psi & \text{cos}\psi & 0\\ 0 & 0 & 1 \end{array}\right]\cdot \left[\begin{array}{ccc} \text{cos}\theta & 0 & \text{sin}\theta \\ 0 & 1 & 0\\ -\text{sin}\theta & 0 & \text{cos}\theta \end{array}\right]\cdot \left[\begin{array}{ccc} 1 & 0 & 0\\ 0 & \text{cos}\phi & -\text{sin}\phi \\ 0 & \text{sin}\phi & \text{cos}\phi \end{array}\right]$$

Rotation matrices contain information about the relative position of two coordinate systems (in terms of Euler angles) so that they can be used to transform any point in one coordinate system to another. The main problem that inertial sensors experience when using these matrices for the expression of their orientation is the appearance of singularities when a gimbal lock takes place. A gimbal lock is the loss of one degree of freedom when the axes of two of the three gimbals are driven into the same place. To avoid these singularities, MTi inertial sensors can provide quaternions [28] as an efficient and non-singular alternative representation of their relative orientation. Quaternions can be interpreted as a rotation *χ* about a unit vector *n*:

(2) $$q=\left(\text{cos}\left(\frac{\chi }{2}\right),n\cdot \text{sin}\left(\frac{\chi }{2}\right)\right)$$

In order to obtain singularity-free rotation matrices from quaternion representation, Equation (3) must be applied:

(3) $$R=\left[\begin{array}{ccc} 2q_{0}^{2}+2q_{1}^{2}-1 & 2q_{1}q_{2}-2q_{0}q_{3} & 2q_{1}q_{3}+2q_{0}q_{2}\\ 2q_{1}q_{2}+2q_{0}q_{3} & 2q_{0}^{2}+2q_{2}^{2}-1 & 2q_{2}q_{3}-2q_{0}q_{1}\\ 2q_{1}q_{3}-2q_{0}q_{2} & 2q_{2}q_{3}+2q_{0}q_{1} & 2q_{0}^{2}+2q_{3}^{2}-1 \end{array}\right]$$

Given (1), sensor roll (*φ*), pitch (*θ*) and yaw (*ψ*) can be obtained by applying the following formulae:

(4) $$\text{roll}={\text{tan}}^{-1}\left(R_{3,2}/R_{3,3}\right)$$

(5) $$\text{pitch}={\text{sin}}^{-1}\left(R_{3,1}\right)$$

(6) $$\text{yaw}={\text{tan}}^{-1}\left(R_{2,1}/R_{1,1}\right)$$

Here, the arctangent is the four quadrant inverse tangent function.

The acquisition system is then the combination of the motion capture system and the software module that calculates the corresponding kinematic model DoF. As can be observed in Figure 1, the motion capture system is composed of a set of four inertial sensing units and a sensor controller that, via USB interface, sends orientation data to the processing unit at a pre-programmed rate between 20 and 80 frames per second. After the estimation of the corresponding DoF, the system provides output IP packets containing the biomechanical parameters of therapeutic interest.

The processing unit, displayed in Figure 1, consists of two software modules: one that reconstructs the kinematic model and another that estimates the associated biomechanical parameters. Both of these modules are described in detail in the following sections.

### 2.2. Kinematic Model

Human upper limb motion can be approximated as the articulated motion of rigid body parts [29]. These segments are upper arm (between the shoulder and elbow joints), forearm (between the elbow and wrist joints) and hand (from the wrist joint on). It is important to take into account that, in this work, the back segment is considered for two main reasons: to create independence between calculations and user position (where the user is facing) and to provide a mechanism to consider the compensations of the actual movements by inclination.

Every joint has its own local axis. Shoulder is modeled as a ball and socket joint with three DoF, located in the center of the humeral head. Movements are calculated between the vector representing the humerus and the trunk. Elbow is modeled as rotating hinge joint with two DoF with a single joint in the distal humerus. Finally, wrist is modeled as a single joint with only one DoF, that is calculated between the vector representing the hand and a fixed point representing the center of the wrist (between radial and cubital stiloid espinas).

**Figure 3.**
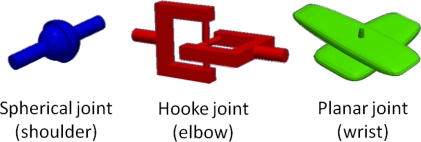
Robot manipulator approach.

Thus, the kinematic chain that this model produces consists of six variables or DoF: three in the shoulder joint (flexion/extension, abduction/adduction and rotation), two in the elbow joint (flexion/extension and pronation/supination) and one in the wrist joint (flexion/extension). In terms of robot manipulators [30], this kinematic model can be approximated as the concatenation of one 3-DoF spherical joint, one 2-DoF Hooke joint and one revolute joint with just one DoF (Figure 3), always considering the human range of movements. It is important to consider at this point that when a manipulator has less than six DoF, it cannot attain general goal positions and orientation in tridimensional space [31].

Given this model, upper limb movement can be represented as the temporal evolution of the six defined degrees of freedom (how the different DoF change over time). It is important to note that, in the present work, relative angular values are provided following the methodology proposed in [32].

The proposed kinematic model includes important simplifications of the actual physiological upper limb:

- Each joint is defined from a joint center. In particular, the shoulder joint is considered as a simple spherical joint that maintains functional shoulder movements but does not preserve the real physiological configuration.
- The forearm is considered as a rigid body, meaning that pronation and supination movements must be considered around the elbow.
- The hand is modeled as a rigid body.

**Figure 4.**
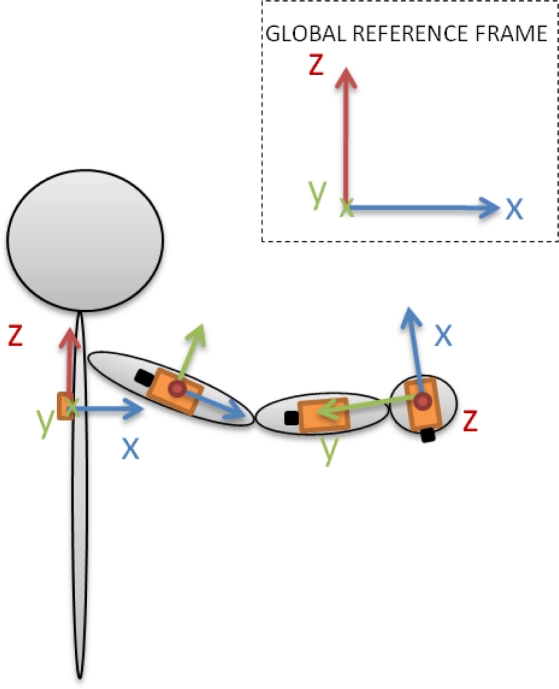
Schematic view of the inertial sensor location.

To represent the defined 6-DoF upper limb model, a set of four inertial sensors has to be mounted on the subject as depicted in Figure 4. This setup consists of one reference sensor attached to the back of the subject (parallel to the scapular spine), one on the upper arm (along the external long head of the triceps), another on the distal forearm (dorsal) and the last one on the dorsal hand surface. In this figure, global and local reference frames can be also observed:

- The global reference frame *z*-axis is defined along the axial axis (from the feet to the head) of the subject, the *x-*axis along the sagittal (from the left shoulder to the right shoulder) axis and the *y-*axis along the coronal axis (from the back to the chest).
- The *y*-axis of the back sensor local frame is defined along the sagittal axis of the subject, the *x-*axis along the coronal axis and the *z-*axis along the axial axis.
- The *x*-axis of the upper arm and forearm sensor local frames are defined along the segment they represent with the *y-*axis parallel to their axis of rotation and the *z-*axis perpendicular to both.
- Finally, the hand sensor is located in such a way that the *y*-axis is defined along the hand segment, the *x-*axis perpendicular over the hand surface and the *z*-axis perpendicular to both.

For the virtual reconstruction of the upper limb, it is necessary to solve a inverse kinematic problem such that the sensor space I is mapped onto the posture space Φ. The solution to this problem consists of determining the biomechanical parameters of interest from the position and orientation of the local frames (attached to the segments) relative to the base frame, that is, the Earth.

Assuming that *R_GS_* is the rotation matrix that rotates a vector in the sensor coordinate system (*S*) to the global reference system (*G*), then:

(7) $$x_{G}=R_{\text{GS}}\cdot x_{S}={\left(R_{\text{SG}}\right)}^{T}\cdot x_{S}$$

Using the above formula and considering the position of the inertial sensor relative to the segment it represents (back segment along the positive *z*-axis, upper arm and forearm along the positive *x*-axis and hand segment along the negative *y*-axis), the reconstruction of the upper limb position is performed as follows:

(8) $$\text{back}=R_{\text{GS}}^{B}\cdot \left(\begin{array}{c} 0\\ 0\\ l_{B} \end{array}\right);\text{arm}=R_{\text{GS}}^{A}\cdot \left(\begin{array}{c} l_{A}\\ 0\\ 0 \end{array}\right);\text{forearm}=R_{\text{GS}}^{F}\cdot \left(\begin{array}{c} l_{F}\\ 0\\ 0 \end{array}\right);\text{hand}=R_{\text{GS}}^{H}\cdot \left(\begin{array}{c} 0\\ -l_{H}\\ 0 \end{array}\right)$$

In the above formulae, $R_{\text{GS}}^{B}$, $R_{\text{GS}}^{A}$, $R_{\text{GS}}^{F}$ and $R_{\text{GS}}^{H}$ represent the 3 × 3 rotation matrices corresponding to the four different sensors (one per model segment), whereas parameters *l_B_*, *l_A_*, *l_F_* and *l_H_* represent the subject anthropometric measurements. The vectors *back*, *arm*, *forearm* and *hand* correspond to the relative upper limb segments in base frame coordinates.

### 2.3. Facing Effect Correction

The first step, prior to the calculation of the degrees of freedom, is to make the system independent of the direction the user is facing. This is performed by means of the data provided by the sensor located on the back surface of the patient.

For facing correction, the following operations must be carried out:

- Creation of a correction rotation matrix with zero roll and the pitch and the yaw equal to the facing coefficients (deviation from the initial calibration that has to be done to align the sensors coordinate frames with the global reference frame).
- Correction of the facing effect by applying an inverse rotation of the upper arm segment rotation using (9):
  (9) $$R_{\text{GS}}^{A^{\text{no}-\text{facing}.-\text{effect}}}=R_{\text{correction}}^{-1}\cdot R_{\text{GS}}^{A}$$

### 2.4. DoF Calculation

#### Shoulder

Because the shoulder joint is the first joint within the upper limb kinematic chain, the three defined DoF can be directly calculated from the upper arm sensor roll, pitch and yaw using Equations (4), (5) and (6) right after the facing effect correction (using $R_{\text{GS}}^{A^{\text{no}-\text{facing}-\text{effect}}}$ matrix) and considering the sensor location relative to the back segment reference frame.

Shoulder flexion/extension (FexS) is directly related to upper arm sensor pitch. Flexion and extension differentiation is made on a 3D coordinate basis: if the upper arm tip is in front of the subject, the movement is flexion (positive value); otherwise, it is extension (negative value). Equation (10) must be applied to match [32]. Note that when the upper arm tip is aligned with the horizontal plane of the shoulder, the flexion/extension takes a value of 90°. This calculation is valid for both left and right arms.

(10) $$\text{flexion}/\text{extension}=\{\begin{array}{c} -\left(\text{pitch}-90^{{}^{\circ}}\right);\text{flexion}\\ \text{pitch}-90^{{}^{\circ}};\text{extension} \end{array}$$

Shoulder abduction/adduction (AbdS) is directly related to upper arm sensor yaw. In the case of the right arm, this DoF corresponds to the upper arm sensor yaw changing its sign, whereas in the case of the left arm, abduction/adduction directly matches sensor yaw. Abduction (separation) and adduction (approaching) differentiation is also made depending on the current position of the upper arm tip in such a way that, if the upper arm tip separates from the subject, the abduction/adduction takes a positive value; on the other hand, if the upper arm tip approaches the subject, the abduction/adduction takes a negative value. When the upper arm tip is aligned with the vertical plane of the shoulder, the abduction/adduction takes a value of 0°.

Shoulder rotation (RotS), assuming that the sensor is perfectly linked to the arm’s bony prominences, can be directly obtained by subtracting 90° from the upper arm sensor roll (roll-90°) in the case of the right arm or subtracting 90° from the minus sensor roll in the case of the left arm (-roll-90°).

#### Elbow

In the case of the elbow joint, it is necessary to proceed to an inverse rotation of the forearm sensor rotation matrix ( $R_{\text{GS}}^{F}$), considering the previous segment (upper arm) in such a way that the data provided by the sensor representing the forearm are not affected by the shoulder joint movements or by the facing effect. This inverse rotation is carried out in the following way:

(11) $$R_{\text{GS}}^{F^{\prime }}=R_{\text{GS}}^{A^{-1}}\cdot R_{\text{GS}}^{F}$$

After the inverse rotation, elbow flexion/extension (FexE) matches sensor yaw in the case of the right arm and minus sensor yaw in the case of the left arm.

Elbow pronation/supination (PronoE) can be obtained from the information provided either by the forearm sensor or by the hand sensor. Ideally, in the case of having the sensors perfectly attached to the bony prominences of the upper limb, the forearm sensor would reflect the entire pronation/supination movement. As this is not the case in the current implementation, the most efficient way of calculating elbow pronation/supination is to use the hand sensor as the main source of information.

First, it is necessary to proceed to an inverse rotation of the hand sensor that considers previous segment configurations (forearm and upper arm) so that the data provided by this sensor are not affected by the shoulder or by the elbow joint movements and the facing effect. This inverse rotation is carried out in the following way:

(12) $$R_{\text{GS}}^{H^{\prime }}=R_{\text{GS}}^{F^{-1}}\cdot R_{\text{GS}}^{H}$$

Elbow pronation/supination corresponds to the addition of the forearm sensor roll and the wrist sensor minus pitch (changing the sign of the global operation in the case of the right arm and keeping it in the case of the left arm) after their corresponding inverse rotations (both values are obtained from $R_{\text{GS}}^{F^{\prime }}$ and $R_{\text{GS}}^{H^{\prime }}$ respectively). The forearm sensor roll is added to the hand sensor pitch to compensate the forearm sensor displacement during a pronation/supination movement, because prior to the hand sensor pitch extraction, the inverse rotation directly affects the pronation/supination value. Elbow pronation values are positive and supination values are negative.

#### Wrist

In the case of the wrist joint, it is also necessary to proceed to an inverse rotation that considers previous segments. This inverse rotation is executed following (12). Wrist flexion/extension (FexW) can be directly obtained from the sensor roll for the left and right arm. Flexion has negative values and extension has positive values.

### 2.5. Experimental Work

**Figure 5.**
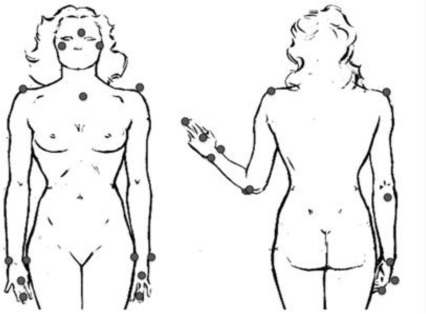
Marker model.

To validate the inertial technology-based motion acquisition system proposed in this paper, BTS SMART-D was used for co-registration. This device is a commercial optoelectronic tracking system used to record DoF from upper limb ADLs. The system consisted of 6 infrared cameras with a recording rate of 140 Hz and two video cameras to register the entire subject’s movement. Smart Capture and Smart Analyzer Software were used. A sixteen-marker model derived from [8] was created for this purpose (Figure 5). The system to be validated is composed of the previously described motion capture system and a test garment where the sensing units are mounted. Since at this point of the research the test garment is not reliable enough (does not keep sensing units positions constant and linked to the upper limb bony prominences), it is important to remark that the aim of this experiment is to validate the proposed DoF calculation methodology, so that in this preliminary validation only one subject has been used.

**Figure 6.**
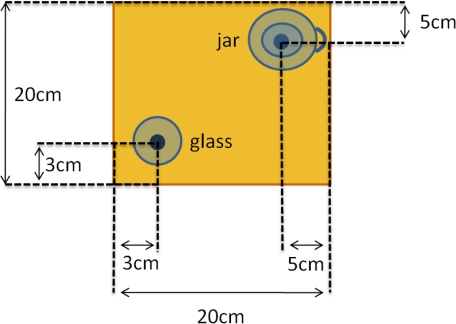
Serving water from a jar ADL setup.

The motions used for validation were the following, both designed by therapists from the Institut Guttmann Neurorehabilitation Hospital:

- Pure movements: shoulder flexion/extension, shoulder horizontal abduction-adduction, shoulder internal rotations, elbow flexion, elbow pronation/supination and wrist flexion-extension (trying not to move the rest of the degrees of freedom).
- Serving water from a jar (setup depicted in Figure 6): a glass jar (with a capacity of 1.5 L) with 150 mL of water was placed to the right (and a bit behind) of the glass (with a capacity of 170 mL). Two solid dots indicate the correct position for the glass and the jar. The subject was asked to fill the glass with the water and leave the jar in the initial position.

**Figure 7.**
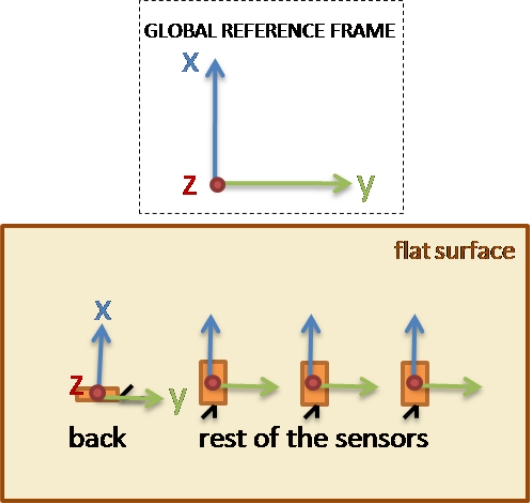
Calibration setup.

Inertial sensors, configured to work with a sampling rate of 50 Hz, that compose the motion acquisition system described in this work need to be calibrated prior to being used. This calibration allows every sensor to align its local reference frame with the global one. After this calibration, all orientation matrices provided by the sensors use the same reference to express their relative orientation. Figure 7 depicts the calibration position of the four sensors from an overhead view.

**Figure 8.**
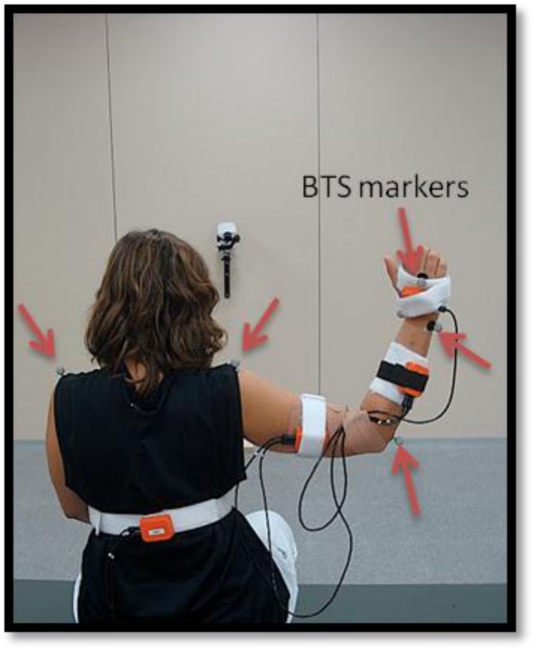
Test garment along with BTS markers for co-registration.

After orientation calibration, sensors were mounted on a test garment worn by the user (Figure 8). This test garment allocates the three sensors mounted on the upper limb in the following positions (back sensor position is not relevant):

- Upper arm sensor: 18 cm from acromion.
- Forearm sensor: 25 cm from epicondyle.
- Hand sensor: 5.5 cm from distal radio-cubital joint.

**Figure 9.**
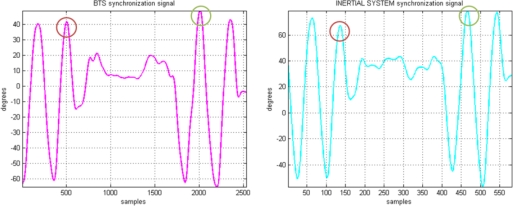
Synchronization signals.

Manual synchronization was done to match the BTS and inertial sensor system signals. For this purpose, at the beginning of each registration, a double wrist flexion-extension movement was performed in such a way that reliable synchronization peaks were present in both signals (see Figure 9, where the circled peaks were used for synchronization).

To perform an accurate comparison of the BTS and inertial systems, signal alignment must be carried out. As there are two synchronization waves in both signals and the sampling frequencies of both systems are known, the following alignment procedure was used:

1. Signal trimming: elimination, in both signals, of the samples before the start synchronization wave and after the end synchronization wave. At this point, although the signals have a different number of samples, the time duration is the same, so the sampling frequency ratio between both signals can be obtained.
2. Resampling of the inertial system signal: as the inertial sensor monitoring system sampling frequency is lower than the BTS, it is necessary to stretch its signal.

Validation data were acquired from one female subject with the following anthropometric measurements:

- Acromion to epicondyle: 35.5 cm.
- Epicondyle to radio-cubital joint: 25 cm.
- Radio-cubital joint to 3rd metatarsal head: 8 cm.

Once the signals have been aligned, the following similarity parameters are calculated to evaluate system performance:

- Correlation coefficient between signals.
- Mean difference between significant peaks in pure movements: the difference between all the significant peaks of the signals is obtained and a mean function is applied (this measurement is used only in pure movements because their signals basically consist in successive peaks).
- Mean difference along the whole signals in ADL motions.

## 3. Results and Discussion

Table 1, where the biomechanical parameters are referred with abbreviations (FexS, AbdS, RotS, FexE, PronoE and FexW), shows the results obtained for pure movement co-registrations. Movements are repeated twice to validate system performance. As can be extracted from the data, the mean correlation coefficient obtained from all the movements is 0.957, meaning that both signals are almost identical in shape for all DoF. From these correlation values, it can be deduced that the mean peak difference is due to the noise generated by the test garment, which does not attach the sensing units to the bony structures of the upper limb under analysis and does not keep sensors’ locations constant. This noise is increased in the case of shoulder rotation, where the sensor, instead of being linked to bony prominences, is linked to the arm flesh and therefore does not reflect the real rotation (the higher differences that take place between the FexS and FexS2 and between AbdS1 and AbdS2 are due to sensor displacement between capture sessions). This error is forward-propagated to the forearm and hand sensor when obtaining pronation-supination measurements, which increases the calculation error.

On the other hand, Table 2 shows the results obtained from the serving water from a jar ADL, which was repeated five times for validation purposes. As in the previous case, high correlation coefficients were obtained for all DoFs (a total mean of 0.93) so that the measurements provided by the inertial technology-based motion acquisition system and those provided by the BTS are almost identical. In this case, the mean difference between the signals of both devices is also due to garment noise and is more evident in shoulder rotation.

The obtained results are very promising as all the problems that have been found can be associated with the test garment used in the co-registration sessions. These errors can be avoided by the combination of two different but related areas of research:

- Creation of a test garment that maintains the linkage of sensors to the upper limb bony prominences so that noise due to the misalignment of the sensors and the different bony structures can be minimized.
- Modeling of the error that the garment introduces so that post-processing can be applied to the calculations proposed in this paper.

As previously commented, the lack of upper arm sensor movement while the shoulder rotates can be modeled to reduce the error that this effect introduces. This modeling depends not only on the garment but also on the rehabilitation session in case the garment does not guarantee constant sensor positions every time it is used. For minimizing this error, a calibration procedure was designed. First, a full-range shoulder rotation movement must be registered using BTS to generate a normalized pattern of the movement to calibrate (note that this calibration signal is valid for all calibrations in the future so BTS device is not necessary anymore); this signal will be obtained only once as it can be used for all future calibration. To have more reliable and consistent information, it is recommended that this calibration signal be created from data acquired from multiple subjects. Once the calibration signal is ready, the subject, wearing the garment with the inertial sensors mounted on it, performs the same movement (full-range shoulder rotation) so that both signals can be aligned and a transfer function can be derived. This transfer function contains all of the information needed to eliminate the noise introduced by both the misalignment between sensors and bones and the effects of having the sensor on the arm surface instead of being linked to the bony prominences in shoulder rotation movements. The main limitation of this method is that it does not consider the movement of the sensing units while certain motion is being performed (intra-session sensors displacements); nevertheless, this problem can be partially solved by having a garment that keeps the sensors fixed to the initial linkage points. Muscle movements, that also affect sensor relative position, are not modeled by the present calibration method so some residual error will still be present.

In this way, if the proposed calibration procedure is performed before the rehabilitation session begins, garment noise will be minimized. The minimization of the shoulder rotation calculation error will also have an effect on the elbow pronation-supination calculation because the error will not be forward-propagated.

**Figure 10.**
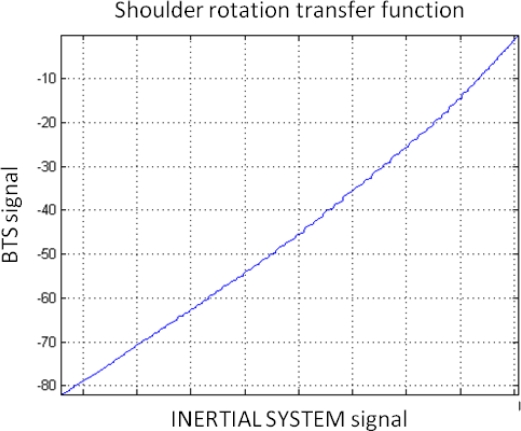
Transfer function that models test garment noise (modeled with a second-order polynomial function).

**Figure 11.**
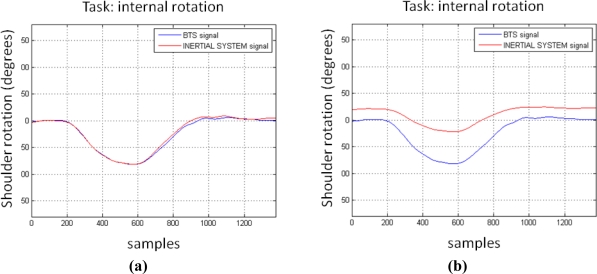
**(a)** Internal rotation signals provided by both systems after calibration. **(b)** Internal rotation signals provided by both systems without calibration.

For the data and the test garment described in the current study, a previous calibration procedure was applied to pure internal rotation movements. Table 3 shows the results obtained for two pure shoulder internal rotations after a calibration procedure was carried out. In this case, the subject performed a full shoulder internal rotation movement immediately before the co-registration so that the transfer function depicted by Figure 10 could be obtained and applied (in this case, this transfer function can be modeled with a second-order polynomial function). After these co-registrations, a marked increase in the calculation performance can be observed as both signals (in both cases) are almost identical (Figure 11).

Figure 11 shows BTS and inertial system shoulder rotation signals when an internal rotation movement was performed. It can be observed that, after applying a transformation following the transfer function displayed in Figure 10, both signals are almost identical. It is important to take into account at this point that this transfer function is subject- and session-dependent.

## 4. Conclusions

In this work, a portable upper limb acquisition system based on inertial technology is proposed. For this purpose, an upper limb kinematic model is defined along with a methodology for calculating the six degrees of freedom associated with the model.

Due to its modular design, the system is completely scalable by changing only the input interface. In this way, if any other sensing hardware is able to provide the same information as the inertial sensors used in this work, no major changes are needed for the rest of the system to obtain the same results.

The fact that the system provides IP packets containing the same calculated biomechanical parameters as output, also makes it independent from the rest of the rehabilitation system, meaning that this motion acquisition system is not only portable in terms of mobility, but also in terms of compatibility with other equipment.

Preliminary validation data demonstrate the accuracy of the inertial technology-based motion analysis system proposed in this paper. A very high correlation was found between the inertial system and validation signals obtained from the BTS SMART-D. The difference between signals is mainly due to the mounting location of the sensors on the test garment; the present study showed how an appropriate calibration methodology can overcome these limitations.

Future work mainly will address the full validation of the shoulder rotation calibration methodology in ADL motions and the creation of a smart garment that is able to minimize the effects of sensor misplacements. Also, the integration of the motion analysis system within a system visualization interface that allows therapists to evaluate user motions in real-time will be researched.

In addition, future work will focus on the integration of the inertial technology-based motion acquisition device with a feedback system that, by comparing the acquired signal with the available motion models, provides users with feedback about their performance so that they can adapt their trajectory in real-time.

## Figures and Tables

**Table 1.** Results of pure movement co-registrations.

	**Correlation**	**Mean peak diff. (degrees)**
**FexS**	0.997	13.2
**FexS 2**	0.992	13.6
**Mean**	0.994	13.4
**AbdS**	0.895	13.6
**AbdS 2**	0.842	20.9
**Mean**	0.718	17.25
**RotS**	0.994	61
**RotS 2**	0.995	59.9
**Mean**	0.995	60.45
**FexE**	0.992	10
**FexE 2**	0.976	1.6
**Mean**	0.984	5.8
**PronoE**	0.962	24.5
**PronoE 2**	0.974	23.7
**Mean**	0.968	24.1
**FexW**	0.980	10.8
**FexW 2**	0.995	12.5
**Mean**	0.987	11.65

**Table 2.** Results of serving from a jar co-registrations.

	**Shoulder flex-ext**		**Shoulder abd-add**		**Shoulder rotation**		**Elbow flex-ext**		**Elbow prono-sup**		**Wrist flex-ext**	
	**Corr.**	**Mean diff. (degrees)**	**Corr.**	**Mean diff. (degrees)**	**Corr.**	**Mean diff. (degrees)**	**Corr.**	**Mean diff. (degrees)**	**Corr.**	**Mean diff. (degrees)**	**Corr.**	**Mean diff. (degrees)**
**Jar 1**	0.998	13.5	0.905	6.3	0.849	28	0.980	18.8	0.960	10.6	0.960	25.7
**Jar 2**	0.992	14.6	0.909	8.5	0.884	27.2	0.982	17	0.840	13.3	0.897	27.2
**Jar 3**	0.995	13.8	0.895	7.8	0.830	28.1	0.977	19.3	0.956	10.8	0.916	26.1
**Jar 4**	0.995	14.2	0.909	7	0.877	29.3	0.977	18.8	0.942	11.4	0.897	29.8
**Jar 5**	0.996	13	0.921	7.6	0.824	31.8	0.980	19.1	0.927	12.4	0.948	25.6
**Mean**	0.995	13.82	0.908	7.44	0.853	28.88	0.979	18.6	0.925	11.7	0.924	26.88

**Table 3.** Results after calibration for pure internal rotation movements.

	**Correlation**	**Mean peak diff. (degrees)**	**Mean peak diff. no calibration (degrees)**
**RotS**	0.998	0.27	60.9
**RotS 2**	0.996	0.81	59.9
**Mean**	0.997	0.54	60.4
